# Objective assessment of a relative afferent pupillary defect by B-mode ultrasound

**DOI:** 10.1371/journal.pone.0202774

**Published:** 2018-08-27

**Authors:** Felix A. Schmidt, Florian Connolly, Matthew B. Maas, Ulrike Grittner, Lutz Harms, Alexander Brandt, Friedemann Paul, Stephan Schreiber, Klemens Ruprecht

**Affiliations:** 1 Department of Neurology, Charité – Universitätsmedizin Berlin, corporate member of Freie Universität Berlin, Humboldt-Universität zu Berlin, and Berlin Institute of Health, Neurocure Cluster of Excellence, Berlin, Germany; 2 NeuroCure Clinical Research Center, Charité – Universitätsmedizin Berlin, corporate member of Freie Universität Berlin, Humboldt-Universität zu Berlin, and Berlin Institute of Health, Neurocure Cluster of Excellence, Berlin, Germany; 3 Berlin Institute of Health (BIH), Berlin, Germany; 4 Department of Neurology, Feinberg School of Medicine, Northwestern University, Chicago, Illinois, United States of America; 5 Department of Biostatistics and Clinical Epidemiology, Charité – Universitätsmedizin Berlin, corporate member of Freie Universität Berlin, Humboldt-Universität zu Berlin, and Berlin Institute of Health, Neurocure Cluster of Excellence, Berlin, Germany; 6 Experimental and Clinical Research Center, Max Delbrueck Center for Molecular Medicine and Charité – Universitätsmedizin Berlin, Berlin, Germany; 7 Department of Neurology, Asklepios Fachklinikum Brandenburg, Brandenburg an der Havel, Germany; LV Prasad Eye Institute, INDIA

## Abstract

**Purpose:**

To evaluate B-mode ultrasound as a novel method for objective and quantitative assessment of a relative afferent pupillary defect (RAPD) in a prospective case-control study.

**Methods:**

Seventeen patients with unilateral optic neuropathy and a clinically detectable RAPD and 17 age and sex matched healthy controls were examined with B-mode ultrasound using an Esaote-Mylab25 system according to current guidelines for orbital insonation. The swinging flashlight test was performed during ultrasound assessment with a standardized light stimulus using a penlight.

**Results:**

B-mode ultrasound RAPD examination was doable in approximately 5 minutes only and was well tolerated by all participants. Compared to the unaffected contralateral eyes, eyes with RAPD showed lower absolute constriction amplitude of the pupillary diameter (mean [SD] 0.8 [0.4] vs. 2.1 [0.4] mm; *p* = 0.009) and a longer pupillary constriction time after ipsilateral light stimulus (mean [SD] 1240 [180] vs. 710 [200] ms; *p* = 0.008). In eyes affected by RAPD, visual acuity correlated with the absolute constriction amplitude (*r* = 0.75, *p* = 0.001).

**Conclusions:**

B-mode ultrasound enables fast, easy and objective quantification of a RAPD and can thus be applied in clinical practice to document a RAPD.

## Introduction

A relative afferent pupillary defect (RAPD) is an impairment of the pupillary light reflex (PLR) upon an ipsilateral light stimulus, typically due to ipsilateral optic nerve dysfunction [1]. At the bedside, presence of a RAPD is commonly tested by the swinging flashlight test, during which a penlight is swung from the unaffected eye to the affected eye while the PLR is observed. In pathological conditions, the pupil of the affected eye constricts normally following a contralateral (indirect) light stimulus (L_stim_), but shows a reduced or absent constriction, or even a paradoxical dilatation, following an ipsilateral (direct) L_stim_ [2]. The most common underlying pathology of RAPD is optic neuritis (ON), which can occur in a variety of neuroimmunological disorders such as multiple sclerosis, neuromyelitis optica spectrum disorder, myelin oligodendrocyte glycoprotein antibody associated encephalomyelitis or sarcoidosis [3–7]. While, in our center, B-mode ultrasound is an established method for quantitatively measuring pupil diameter [8], the role of B-mode ultrasound in the assessment of a RAPD has not been systematically studied so far. Here, we evaluate B-mode ultrasound for objective and quantitative assessment of a RAPD.

## Material and methods

### Study participants

In this prospective case-control study, study participants were recruited from the Department of Neurology and from the NeuroCure Clinical Research Center, Charité–Universitätsmedizin Berlin. Inclusion criteria were age 18 to 65 years, and a clinically detectable RAPD due to unilateral ON. RAPD was diagnosed clinically by bedside examination with the swinging flashlight test by a trained physician [2]. We excluded patients with a history of any ocular disease other than ON (e.g. glaucoma, cataract, macular degeneration or diabetic retinopathy), those who had undergone any type of ocular surgery in the past including laser surgery, and those taking any topical or systemic medications known to potentially affect pupillary function. Data from healthy controls were taken from a previous study on B mode ultrasound assessment of the PLR in healthy individuals [8]. Healthy controls had no prior or current ophthalmologic disease, no medications known to potentially affect pupillary function, and were selected from our database to match patients for age (± 1 years) and sex.

### Visual acuity testing

Habitual corrected visual acuity was determined under standardized light conditions using a Snellen Chart with a distance of 2.8 meter [9].

### B-mode ultrasound technique

All participants were studied in supine position under standardized dimmed light conditions (room lighting 30 Lux). To adapt to the light level, study participants spent at least 10 minutes in the ultrasound examination room before testing. All insonations were performed by the same investigator (SJS) with the subject’s eyes closed using an Esaote Mylab 25 system (Esaote, Indianapolis, USA) equipped with a linear 10 MHz probe. Power settings were reduced to minimum, according to the ALARA (as low as reasonably achievable) insonation approach and current guidelines for orbital insonation [10]. B-mode settings were adjusted for near-field eye examination. Each patient was examined lying flatly on the examination bed facing towards the ceiling. Each pupil was visualized with the probe gently positioned at an angle of 20–30 degree from the examination bed on the lower eyelid of the closed eye, leveraging Bell’s phenomenon [8]. For assessment of the PLR, patients had their eyes closed, a penlight was activated approximately 2 cm in front of each closed eye and the light reaction of each pupil was digitally documented. In every examination the same standard penlight was used with a luminous emittance of 70,000 Lux and a stimulus time of 2 seconds to ensure constant wavelength, intensity and duration of the light stimulus. Each assessment was performed in exactly the same order, starting by measuring the pupillary diameter (PD) of the left eye at rest as well as during ipsilateral and contralateral L_stim_, followed by the same examinations of the right eye.

### Data analyses

RAPDs were documented by recording video sequences of ultrasound examinations of the PLR during performance of the swinging flashlight test [2]. PDs were manually assessed in a frozen still image of the pupil, which was then digitally stored. Using the measuring tool of the native application ultrasound system software, the largest diameter at rest and the smallest diameter after L_stim_ were measured. Pupillary constriction time (PCT, measured in milliseconds) was defined as the time interval between the maximum and the minimum PD during L_stim_. PCT was manually determined using the AVSVideoConverter9.2 freeware (Online Media Technologies Ltd. London, UK) in recorded 5 second video sequences of a second ipsi- and contralateral L_stim_, approximately 2 min after the PD analysis.

### Statistical analyses

Statistical analyses were performed using SPSS 23.0 (IBM SPSS Statistics, New York, USA). Graphs were created with GraphPadPrism 7.0 (GraphPad Software Inc, La Jola, USA). PD and PCT are reported as mean ± standard deviation. PLR assessment measures in patients with RAPD were compared with same-sided eyes of healthy controls using Student´s t-test for independent samples for continuous variables or Mann-Whitney-U-test, depending on the distribution. Differences between affected and non-affected eyes in patients were tested using Wilcoxon signed-rank-test. Associations between visual acuity and PLR assessment measures were analyzed using Pearson’s correlation coefficient. We also calculated the ratio of the consensual/direct absolute constriction amplitude for each eye to quantitatively express the severity of the RAPD (“constriction ratio”). Correlation of the constriction ratio with visual acuity was analyzed using Spearman’s rank correlation coefficient. P-values were corrected for multiple testing according to the Bonferroni method [11]. Thus, each p-value was multiplied by the number of statistical tests performed (n = 30). A two-sided significance level of α = 0.05 was considered significant.

The study was approved by the institutional review board of Charité—Universitätsmedizin Berlin (EA1/190/15) and all participants provided written informed consent.

## Results

### Study participants

We studied 17 patients with unilateral ON and a clinically detectable RAPD. Of these, 5 had ON as a clinically isolated syndrome (CIS), 4 had relapsing remitting multiple sclerosis according to the McDonald 2010 criteria [12], 6 had neuromyelitis optica spectrum disorder according to the Wingerchuk 2015 criteria [13], and 2 had ON of unknown etiology. The demographics and clinical characteristics of the patients and controls are summarized in Table 1.

**Table 1:** Demographics and clinical characteristics.

	Patients with ON (n = 17)	Healthy Controls (n = 17)
**Age (years)**		
mean (standard deviation)	43 (13)	43 (13)
**Sex**		
Female/male	12/5	12/5
**Time since ON onset (days)**		
median (minimum—maximum)	50 (5–5475)	n.a.

ON = optic neuropathy, n.a. = not applicable

In patients with a RAPD, the visual acuity of affected eyes was significantly lower compared to the same-sided eyes of controls (mean [SD] 0.4 [0.2] vs. 1.0 [0.1]; *p*<0.001) and compared to the unaffected fellow eyes (0.4 [0.2] vs. 0.9 [0.1]; *p* = 0.008) (Table 2).

**Table 2:** Visual acuity testing and ultrasound assessment.

	Affected eye (n = 17)	Unaffected eye (n = 17)	HC same-sided as affected eye (n = 17)	HC same-sided as unaffected eye (n = 17)	*p*-value^a^ (Affected eye vs. HC)	*p*-value^a^ (Unaffected eye vs. HC)	*p*-value^a^ (Affected vs. Unaffected eye)
Visual Acuity							
Decimal	0.4 (0.2)	0.9 (0.1)	1.0 (0.1)	1 (0.1)	**<0.001**	> 0.999	**0.008**
PD at rest							
(mm)	4.8 (0.7)	5.1 (0.6)	4.7 (0.9)	4.6 (0.9)	> 0.999	> 0.999	0.390
PD during ipsilateral light stimulus							
(mm)	4.0 (0.7)	3.0 (0.4)	2.6 (0.6)	2.8 (0.7)	**<0.001**	> 0.999	**0.008**
Difference between PD at rest and during ipsilateral light stimulus (direct constriction amplitude)							
(mm)	0.8 (0.4)	2.1 (0.4)	2.0 (0.5)	1.9 (0.3)	**<0.001**	> 0.999	**0.009**
PD during contralateral light stimulus							
(mm)	3.0 (0.6)	4.0 (0.7)	2.5 (0.6)	2.7 (0.6)	0.720	**<0.001**	**0.009**
Difference between PD at rest and during contralateral light stimulus (consensual constriction amplitude)							
(mm)	1.9 (0.5)	1.1 (0.5)	2.0 (0.5)	1.9 (0.4)	> 0.999	**<0.001**	**0.019**
Pupillary constriction time after ipsilateral light stimulus							
(ms)	1240 (180)	710 (200)	830 (130)	880 (183)	**<0.001**	0.800	**0.008**
Pupillary constriction time after contralateral light stimulus							
(ms)	750 (190)	1080 (250)	850 (120)	890 (100)	> 0.999	**<0.001**	**0.007**
RAPD assessment							
Pos./Neg.	17/0	0/17	0/17	0/17	--	--	--
Constriction ratio of consensual to direct constriction amplitudes							
	2.8 (1.7)	0.6 (0.2)	1.0 (0.1)	1.0 (0.1)	**<0.001**	**<0.001**	**0.009**

Values are given as mean ± standard deviation. HC = healthy controls, n = number, neg. = negative, pos. = positive, PD = pupillary diameter, RAPD = relative afferent pupillary defect.

^a^Bonferroni-corrected *p*-values according to the number of tests (n = 30).

### B-mode ultrasound for assessment of RAPD

The average ultrasound examination was doable in approximately five minutes and was well tolerated by all participants. In all 17 patients with a clinically detectable RAPD, RAPD was also detectable with B-mode ultrasound. Results of visual acuity testing, pupil diameter and PCT assessment are summarized in Table 2. For illustration of RAPD assessment with B-mode ultrasound, a video sequence of a 42-year-old male with left ON due to multiple sclerosis is provided as supplementary video material (S1 Video).

### Absolute constriction amplitudes

During ipsilateral L_stim_ of the affected eye the direct constriction amplitude was smaller in the affected eyes compared to the same-sided eyes of controls (mean [SD] 0.8 [0.4] vs. 2.0 [0.5] mm; *p*<0.001) and compared to the unaffected fellow eyes (0.8 [0.4] vs. 2.1 [0.4] mm; *p* = 0.009) (Table 2). Consequently, during ipsilateral L_stim_ of the affected eye the consensual constriction amplitude in the unaffected eye was smaller compared to same-sided eyes of controls (mean [SD] 1.1 [0.5] vs. 1.9 [0.4] mm; p<0.001) and compared to the consensual constriction amplitude of affected eyes (1.1 [0.5] vs. 1.9 [0.5]; p = 0.019) (Table 2).

### Constriction ratio

**Fig 1:**
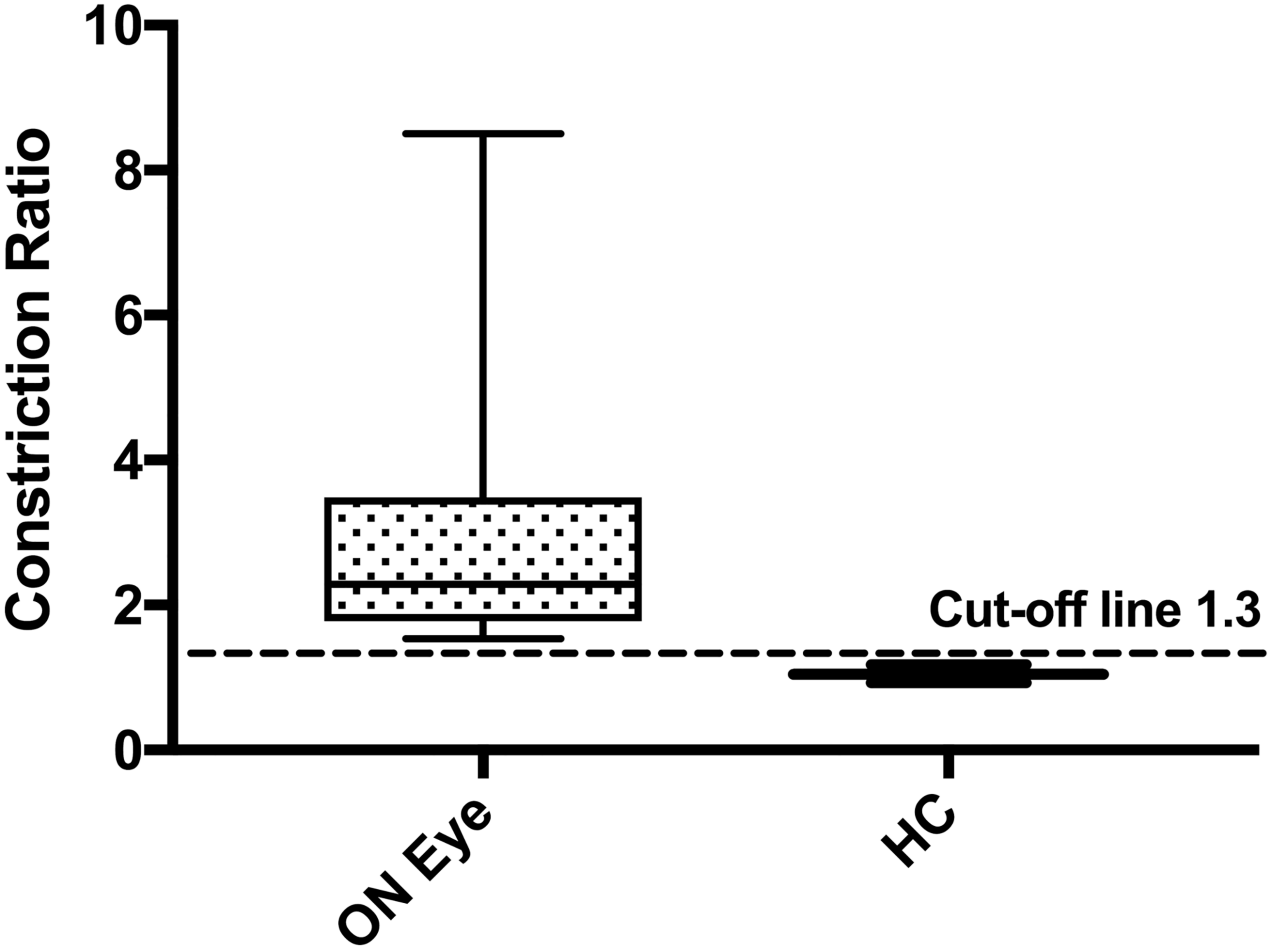
Comparison of constriction ratio of consensual to direct constriction amplitude between affected and HC eyes. X-axis: affected eyes and HC eyes. Y-axis: Constriction ratio of consensual to direct constriction amplitudes; the whiskers indicate minimum and maximum values. HC = healthy controls, ON = optic neuritis.

As a quantitative measure of RAPD severity we calculated the constriction ratio, defined as the quotient of the consensual to direct constriction amplitudes. The constriction ratio was higher in the affected eyes than in the same-sided eyes of controls (mean [SD] 2.8 [1.7] vs 1.0 [0.1]; *p*<0.001) and lower in the unaffected fellow eyes compared to controls (0.6 [0.2] vs. 1.0 [0.1]; *p*<0.001) (Table 2). To establish a cut-off value that distinguishes between pathological and normal ratios, we calculated the mean + 3 standard deviations of the constriction ratio of the control eyes (i.e. 1.3) and defined values above this value as pathological. As shown in Fig 1, a threshold value of >1.3 discriminated eyes with a clinically-defined RAPD and healthy control eyes without any overlap, suggesting that this measure could be used in clinical practice for a diagnosis of a RAPD by B-mode ultrasound.

### Pupillary constriction times

During ipsilateral L_stim_, PCT was longer in the affected eyes compared to the same-sided eyes of controls (mean [SD] 1240 [180] vs. 830 [130] ms; *p*<0.001) and compared to the unaffected fellow eyes (1240 [180] vs. 710 [200] ms; *p* = 0.008). During contralateral L_stim_, PCT was longer in the unaffected eyes compared to the same-sided control eyes (mean [SD] 1080 [250] vs. 890 [100] ms; *p*<0.001) and compared to affected eyes (1080 [250] vs. 750 [190] ms; p = 0.007).

### Correlation of visual acuity with pupillary constriction

**Fig 2:**
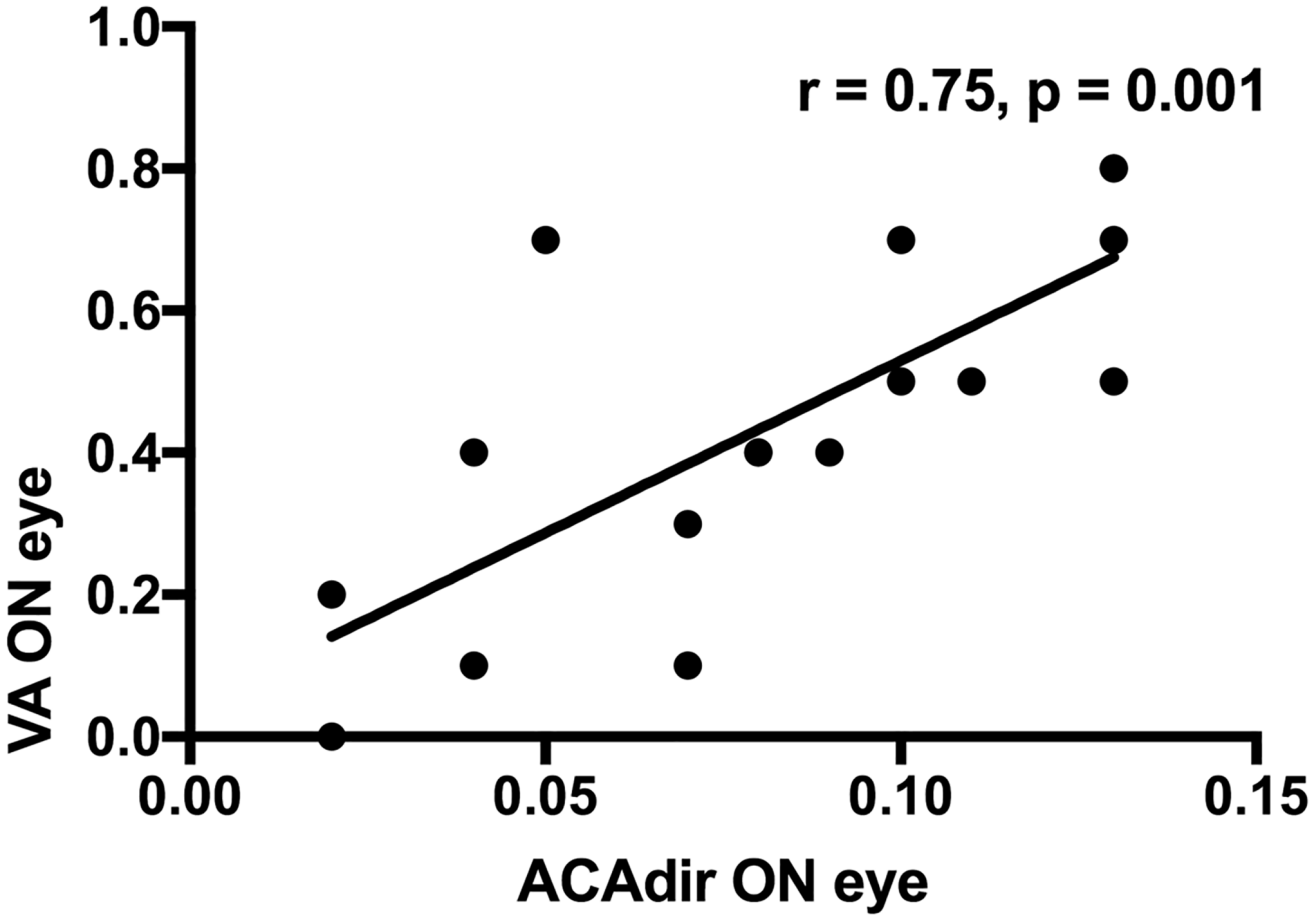
Correlation of visual acuity with absolute constriction amplitude. X-axis: ACA_dir_ = difference between pupillary diameter at rest and during ipsilateral light stimulus, ON = optic neuritis. Y-axis: VA = visual acuity.

The visual acuity of the affected eyes was correlated with the direct constriction amplitude of the affected eye (*r* = 0.75, *p* = 0.001, Fig 2) and with the consensual constriction amplitude of the contralateral eye (*r* = 0.74, *p* = 0.001). The visual acuity of the affected eye was inversely correlated with the constriction ratio of the affected eye (*r* = -0.66, *p* = 0.004).

## Discussion

We here report on the application of B-mode ultrasound as a novel method for the detection and quantification of a RAPD. B-mode ultrasound assessment of RAPD was fast, simple and well–tolerated, and enabled unambiguous detection of a RAPD in patients with a clinically detectable RAPD due to unilateral ON. As expected, ultrasound measurements showed reduced constriction amplitudes in both eyes following light stimulation of the affected eye compared to contralateral light stimulation. Furthermore, the severity of the RAPD as assessed by B-mode ultrasound correlated with the severity of visual acuity impairment.

Compared to standard clinical RAPD examination by the swinging flashlight test [1, 2], advantages of RAPD testing by B-mode ultrasound include objective quantification of the severity of a RAPD and digital documentation of results of RAPD examinations. Parameters and ultrasound images can thus be stored and analyzed longitudinally in follow-up measurements. Furthermore, the dynamic component of the PLR, the PCT, can be measured and documented as well.

The results for constriction amplitude differences obtained by B-mode ultrasound were overall similar to those of studies examining patients with RAPD and controls with infrared video pupillometry (IVP) [14–17]. While IVP allows for a marginally more detailed analysis of the PLR [18], IVP devices are sophisticated tools with limited availability due to high acquisitions costs. In contrast, ultrasound is a widely available standard diagnostic tool in most hospitals and medical practices. Furthermore, unlike IVP, PLR assessment by ultrasound can be performed with the patient´s eyes closed, so examinations are still feasible in cases in which eyelid retraction is impeded.

To distinguish in clinical routine between healthy and pathological reactions, we propose a threshold value of >1.3 of the consensual to direct constriction amplitude ratio as suggestive of RAPD. To determine this threshold, we applied the “mean + 3 standard deviation of negative controls” formula, a widely used formula to determine cut-offs for biological tests [19]. The constriction ratio can be measured with B-mode ultrasound within approximately 2 min and could help to identify a RAPD when it is not clearly detectable with the swinging flashlight test [2]. Furthermore, constriction ratios could be longitudinally evaluated in follow-up examinations to document RAPD severity and to monitor treatment effects in patients with inflammatory optic neuropathies. Determination of an RAPD by B-mode ultrasound can measure functional integrity of the anterior visual pathway, whereas optical coherence tomography measures structural damage of the retina, and visual evoked potentials measure functional integrity of the entire visual pathway. B-mode ultrasound of the eye could thus complement visual evoked potentials [6,20] and optical coherence tomography [21–24], in the assessment of the visual pathway and could thus help to investigate afferent anterior visual pathway damage in patients with inflammatory conditions, such as CIS, MS, NMOSD or MOG antibody associated encephalomyelitis [25–29].

Of note, our findings may also have implications for treatment trials with visual endpoints. For instance, one of the recent NMOSD trials investigating the clinical efficiency of a CD19 monoclonal antibody has implemented RAPD as an attack criterion [30]. Similar trials could potentially benefit from a reliable and reproducible RAPD evaluation method such as the B-mode ultrasound proposed here. Thus, longitudinal studies with follow-up measurements of RAPD by ultrasound and their correlation to visual acuity and treatment response would be of interest. Furthermore, it will be interesting to evaluate in patients with ON and no clinical sign of RAPD whether subclinical RAPD can be detected by ultrasound.

Altogether, B-mode ultrasound enables fast, easy and objective quantification of a RAPD and can thus be applied in clinical practice to document a RAPD.

## Supporting information

S1 Video (MP4)
